# Genome-wide identification and comparative analysis of the heat shock transcription factor family in Chinese white pear (*Pyrus bretschneideri*) and five other Rosaceae species

**DOI:** 10.1186/s12870-014-0401-5

**Published:** 2015-01-21

**Authors:** Xin Qiao, Meng Li, Leiting Li, Hao Yin, Juyou Wu, Shaoling Zhang

**Affiliations:** College of Horticulture, State Key Laboratory of Crop Genetics and Germplasm Enhancement, Nanjing Agricultural University, Nanjing, 210095 China

**Keywords:** Hsf, Stress-response, Evolution, Transcriptome sequencing, Pear, Rosaceae

## Abstract

**Background:**

Heat shock transcription factors (Hsfs), which act as important transcriptional regulatory proteins in eukaryotes, play a central role in controlling the expression of heat-responsive genes. At present, the genomes of Chinese white pear (‘Dangshansuli’) and five other Rosaceae fruit crops have been fully sequenced. However, information about the *Hsfs* gene family in these Rosaceae species is limited, and the evolutionary history of the *Hsfs* gene family also remains unresolved.

**Results:**

In this study, 137 *Hsf* genes were identified from six Rosaceae species (*Pyrus bretschneideri*, *Malus × domestica*, *Prunus persica*, *Fragaria vesca*, *Prunus mume*, and *Pyrus communis*), 29 of which came from Chinese white pear, designated as *PbHsf*. Based on the structural characteristics and phylogenetic analysis of these sequences, the *Hsf* family genes could be classified into three main groups (classes A, B, and C). Segmental and dispersed duplications were the primary forces underlying *Hsf* gene family expansion in the Rosaceae. Most of the *PbHsf* duplicated gene pairs were dated back to the recent whole-genome duplication (WGD, 30–45 million years ago (MYA)). Purifying selection also played a critical role in the evolution of *Hsf* genes. Transcriptome data demonstrated that the expression levels of the *PbHsf* genes were widely different. Six *PbHsf* genes were upregulated in fruit under naturally increased temperature.

**Conclusion:**

A comprehensive analysis of *Hsf* genes was performed in six Rosaceae species, and 137 full length *Hsf* genes were identified. The results presented here will undoubtedly be useful for better understanding the complexity of the *Hsf* gene family and will facilitate functional characterization in future studies.

**Electronic supplementary material:**

The online version of this article (doi:10.1186/s12870-014-0401-5) contains supplementary material, which is available to authorized users.

## Background

Plant development and agricultural production are seriously disturbed by adverse environmental conditions such as cold, drought, and excess heat. Heat stress due to increases in temperature beyond a threshold level cause significant damage to plant morphology, physiology, and biochemistry and may drastically reduce plant biomass production and economic yield in many areas worldwide [1,2]. In response, plants have developed numerous sophisticated adaptations over the long course of evolution [3]. Plant survival is dependent upon a network of interconnected cellular stress response systems that involve the activation of a wide range of transcriptional factors; this network is challenged by global climate changes such as global warming, which makes heat stress a significant concern [4–7]. As important gene regulators, transcription factors are involved in an array of plant protective mechanisms and cellular stress-response pathways and play an essential role in enhancing the stress tolerance of crop plants [8–13]. Hsfs are particularly involved in the heat stress response, and these products are important regulators in the sensing and signaling of heat stress [13]. Recent studies have also shown that Hsfs are involved in plant growth and development, as well as in responses to other abiotic stresses such as cold, salt, and drought [12–21]. For example, *HsfA1a* acts as the master regulator of the heat stress response in tomato (*Solanum lycopersicum*) [22]; *HsfA2* is the dominant Hsf in tomato and *Arabidopsis* and is also associated with oxidative and drought stress responses [12,19,23]; *HsfA4a* is related to cadmium tolerance in rice (*Oryza sativa*) [21]; and *HsfA9* is involved in embryogenesis and seed maturation in sunflowers and *Arabidopsis* [16–18].

As do many other transcription factors, Hsfs possess a modular structure composed of several structurally and functionally conserved domains. Hsfs share a common core structure composed of an N-terminal DNA binding domain (DBD) and an adjacent bipartite oligomerization domain (HR-A/B) [24,25]. Some Hsfs also include other well-defined domains: a nuclear localization signal (NLS) domain essential for nuclear import, nuclear export signal (NES) domain rich in leucine, and C-terminal activator domain (CTAD) characterized by aromatic (W, F, Y), large hydrophobic (L, I, V), and acidic (E, D) amino acid residues, known as AHA motifs [13,24,26]. Close to the N-terminus, the DBD is the most conserved region of the Hsfs and is composed of an antiparallel four-stranded β-sheet (β1-β2-β3-β4) and a three-helical bundle (H1, H2, and H3). A central helix-turn-helix motif (H2-T-H3) located in the hydrophobic core of this domain specifically binds to the heat shock promoter elements [27]. The HR-A/B domain is characterized by hydrophobic heptad repeats that form a helical coiled-coil structure, which is a prerequisite for high affinity DNA binding and, subsequently, for transcriptional activity. Furthermore, a flexible linker exists between the DBD domain and HR-A/B domain [28].

Differences in the numbers of *Hsf* genes have been widely determined in angiosperms. In contrast to those of other eukaryotes, which possess one to three heat stress *Hsf* genes, the plant *Hsf* gene family contains a striking number of genes, with more than 20 and up to 52 members in any given species [12,29,30]. According to the structural characteristics of their HR-A/B domain and phylogenetic comparisons, plant *Hsf* genes may be divided into three classes: A, B, and C [24,25]. *Hsf* genes of class B are comparatively compact, not containing any insertions, while those of classes A and C have insertions of 21 (class A) and seven (class C) amino acid residues between the A and B components of the HR-A/B domain. This classification is also supported by the flexible linker between the DBD domain and HR-A/B domain (9 to 39, 50 to 78, and 14 to 49 amino acid residues for class A, B, and C *Hsf* genes, respectively) [13,24]. In addition, many plant class A *Hsf* genes have a particular signature domain comprising a combination of an AHA motif with an adjacent NES [13,25].

Because of the vital regulatory functions of *Hsf* genes in plant responses to different stresses and developmental processes [18–20], *Hsf* gene family have been extensively studied in the model plant *Arabidopsis thaliana*, as well as in nonmodel plants such as rice (*Oryza sativa*), poplar (*Populus trichocarpa*), maize (*Zea mays*), apple (*Malus domestica*), etc. [9,13,24,31–33]. In comparison with that in other species, the *Hsf* gene family in the Rosaceae has not been widely examined. Pear is a member of the Rosaceae family and is also the third-most important temperate fruit species [34]. Recently, the genome of the domesticated Chinese white pear (*Pyrus bretschneideri* Rehd. cv. ‘Dangshansuli’) [34] has been fully sequenced. Genome sequences are also available for five other Rosaceae species (apple, peach, strawberry, Chinese plum, and European pear). This information provides an opportunity to further analyze the *Hsf* gene family in Rosaceae species. Therefore, our present study aims to annotate the full-length *Hsf* genes in Chinese white pear and other Rosaceae fruit species, infer their expansion and evolutionary history, explore their heat stress responses as elicited by naturally increased temperature, and provide a relatively complete profile of the *Hsf* gene family in Rosaceae. The results of this work will be useful for revealing the mechanisms of thermotolerance in fruit trees and for improving the tolerance of fruit trees to high-temperature stress, which is becoming more prevalent due to global warming.

## Results

### Identification and classification of *Hsf* genes in the Rosaceae

Two strategies were used to search for members of the Hsfs family in *Pyrus bretschneideri* and five other Rosaceae species: Hidden Markov Model search (HMMsearch) with the Hsf domain HMM profile (PF00447) and BLASTP using Hsf protein sequences from *Arabidopsis thaliana* and *Populus trichocarpa* as queries. A total of 185 candidate *Hsf* genes were identified. We removed six and one *Hsf* genes located in unanchored scaffolds of Chinese white pear and Chinese plum, respectively. A further 40 candidates were removed due to an incomplete DBD domain and loss of the functional HR–A/B domain. One abnormal pear *Hsf* (Pbr013854.1) containing a Really Interesting New Gene (RING) finger domain and a tryptophan-aspartic acid 40 (WD40) domain was also removed. The selection of apple *Hsf* genes was based on recent research results [32]. Consequently, 137 nonredundant and complete *Hsf* genes were surveyed in our study. A total of 29 *Hsf* genes were identified in Chinese white pear(*PbHsf*), 33 in European pear (*PcHsf*), 25 in apple (*MdHsf*), 17 in peach (*PpHsf*), 16 in strawberry (*FvHsf*), and 17 in Chinese plum (*PmHsf*) (Table 1).

**Table 1:** **Genome information and** ***Hsf*** **genes number identified in Rosaceae species**

**Common name**	**Scientific name**	**Chromosome number**	**Release version**	**Genome gene number**	**Identified**	**Gene name prefix**
					***Hsf*** **genes**	
Chinese white pear	*Pyrus bretschneideri*	34	NJAU, v1.0	42341	29 (38)	Pbr
Apple	*Malus domestica*	34	GDR, v1.0	63541	25 (49)	MDP
Peach	*Prunus persica*	16	JGI, v1.0	27864	17 (21)	ppa
Strawberry	*Fragaria vesca*	14	GDR, v1.0	32831	16(16)	gene
Chinese plum	*Prunus mume*	16	BFU, v1.0	31390	17 (20)	Pm
European pear	*Pyrus communis*	34	GDR, v1.0	43419	33 (41)	PCP

In this study we totally investigated six Rosaceae species genomes. NJAU, Nanjing Agricultural Univerisity (http://peargenome.njau.edu.cn/); GDR, Genome Database for Rosaceae (http://www.rosaceae.org/); JGI, Joint Genome Institude (http://www.jgi.doe.gov/); BFU, Beijing Forestry University (http://prunusmumegenome.bjfu.edu.cn/index.jsp). The numbers in parenthesis show gene count before filtering the unanchored and incomplete genes.

**Figure 1:**
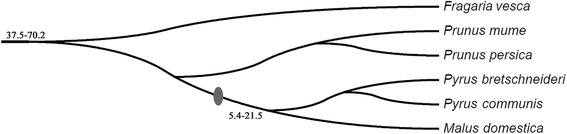
**Species tree of six Rosaceae species.** Solid oval indicates the occurrence of WGD. Numbers in the figure indicate species divergence time. Unit: MYA. The data were downloaded from NCBI Taxonomy common tree (http://www.ncbi.nlm.nih.gov/Taxonomy/CommonTree/wwwcmt.cgi) and the tree was constructed by MEGA6.

The phylogenetic tree of the six Rosaceae species was reconstructed, and the WGD events over the course of genome evolution were inferred from recent studies [34] (Figure 1). Chinese white pear, European pear, and apple belong to the Maloideae, strawberry belongs to Rosoideae, and peach and Chinese plum belong to the Prunoideae [35]. Nearly twice as many *Hsf* genes were present in pear and apple than in peach, strawberry, and Chinese plum. A recent WGD event occurred in the Maloideae but not in the Rosoideae and Prunoideae. We can therefore infer that the recent WGD led to the specific expansion of the *Hsf* gene family in the Maloideae.

**Figure 2:**
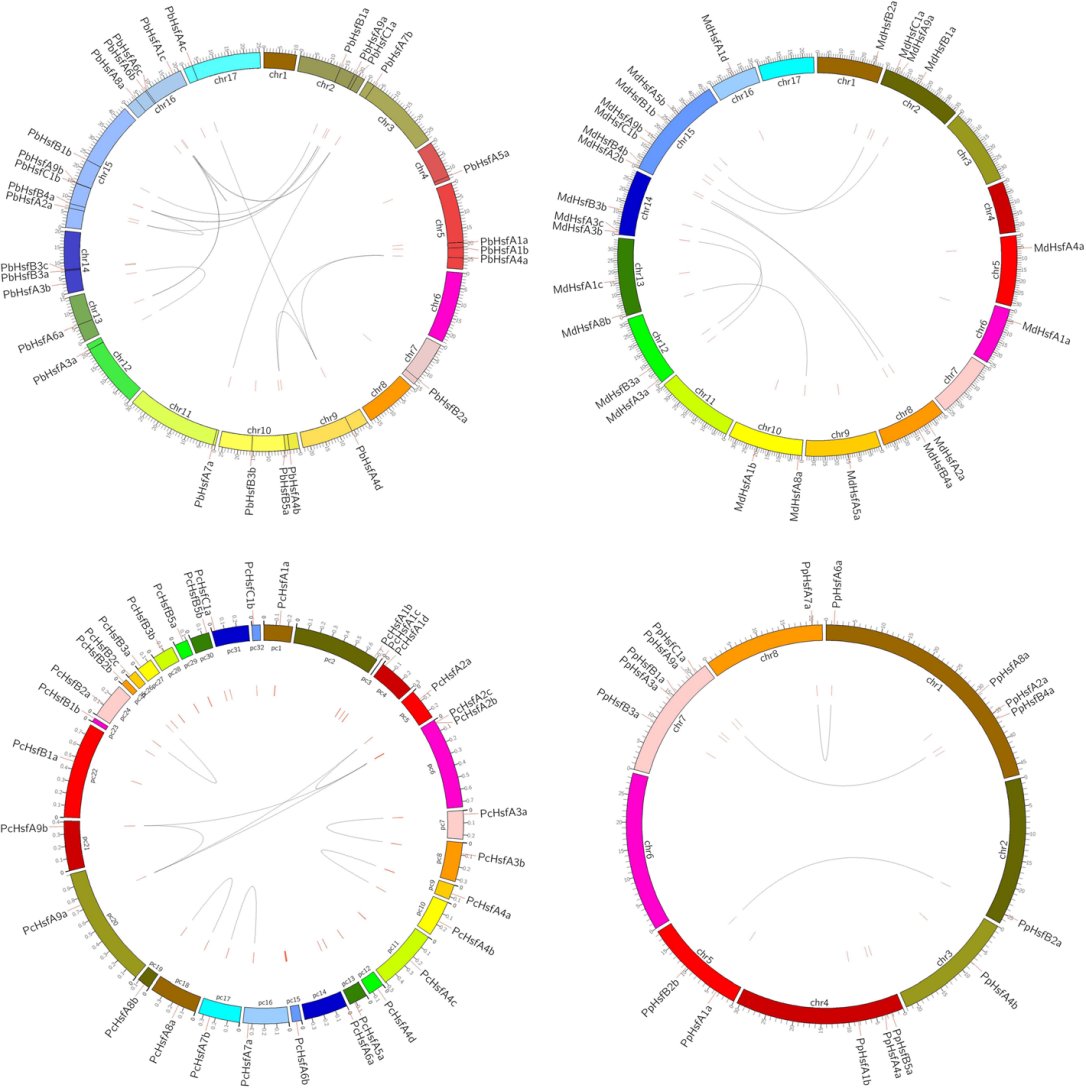
**Localization and synteny of the** ***Hsf*** **genes in Rosaceae genomes.** *Hsf g*enes in Chinese white pear (*PbHsf*), apple (*MdHsf*) and peach (*PpHsf*) were mapped on the different chromosomes, while in European pear (*PcHsf*) were anchored to the scaffolds. Chromosome or scaffold number is indicated on the inner side and highlighted red short lines in the inner circle correspond to different *Hsf* genes. Gene pair with a syntenic relationship was joined by the line.

The *PbHsf* genes are distributed on 14 of the 17 pear chromosomes, with five *Hsf* genes detected on chromosome 15 (Figure 2). Similarly to that in *PbHsf* genes, the distribution of the *Hsf* genes in the other five Rosaceae genomes is random (Figure 2 and Additional file 1).

**Figure 3:**
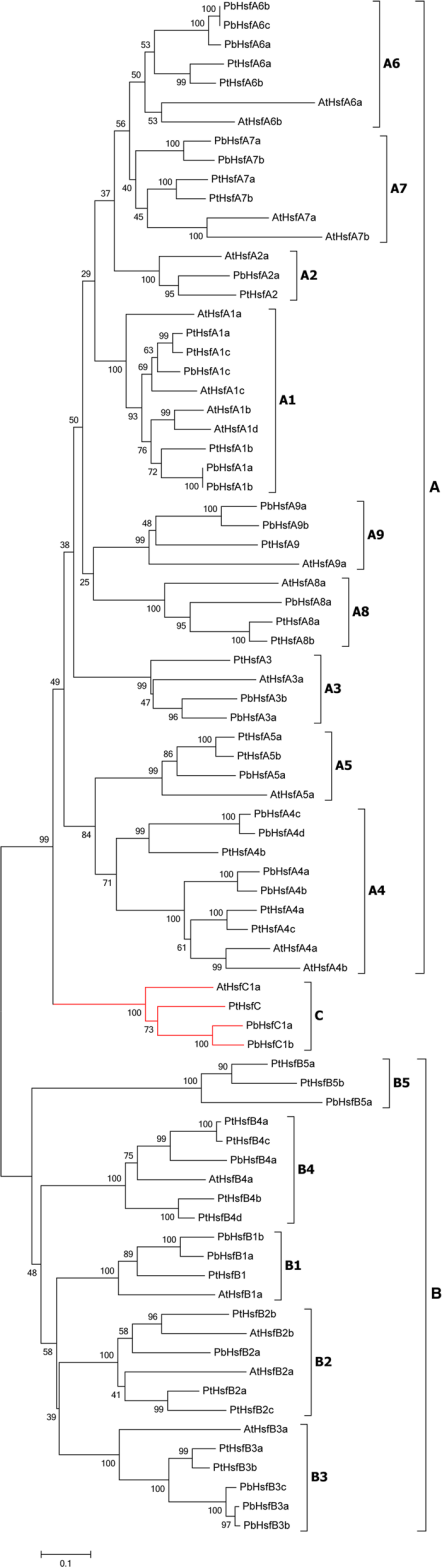
**Neighbor-joining phylogeny of Hsfs from** ***P. bretschneideri*** **,** ***P. trichocarpa*** **and** ***A. thaliana*** **.** The phylogenetic tree was obtained using the MEGA 6.0 software on the basis of amino-acid sequences of the N-terminal domains of Hsfs including the DNA-binding domain, the HR-A/B domain and the linker between these two domains. Bootstrap analysis was conducted with 1000 replicates. The abbreviations of species names are as follows: Pb, *Pyrus bretschneideri*; Pt, *Populus trichocarpa*; At, *Arabidopsis thaliana*.

According to the multiple sequence alignment of the functional domains and the phylogenetic analysis, the members of the Rosaceae *Hsf* family genes were divided into three subfamilies (A, B, and C) (Table 2 and Additional file 2). These results were consistent with the classification of the genes in other plants [24,33]. In contrast with class B, classes A and C possess insertions of amino acid residues in the HR-A/B region. The protein sequences of class A contain more specific domains than do those of class C. Furthermore, a phylogenetic tree was generated using the protein sequences of *Pyrus bretschneideri* (PbHsf), *Populus trichocarpa* (PtHsf), and *Arabidopsis thaliana* (AtHsf) (Figure 3). The tree was constructed using the neighbor joining (NJ) method, and a maximum likelihood (ML) tree confirmed the result. The *Hsf* genes from the three species were clearly grouped into three different clades corresponding to the main *Hsf* classes A, B, and C. In the *PbHsf* genes family, 19, 8, and 2 genes were assigned to Classes A, B, and C, respectively. Within the A clade, nine distinct subclades (A1, A2, A3, A4, A5, A6, A7, A8, and A9) were resolved and contained all of the *PbHsf* genes. The C-type *Hsf* genes from the three plant species also constituted one distinct clade, which appeared to be more closely related to the *Hsf* A-group. Correspondingly, the B-type *Hsf* genes were grouped into a separate clade subdivided into five groups (B1, B2, B3, B4, and B5); notably, the B5 sub-clade was obviously distinct from the other four subclades.

**Table 2:** **Classification of** ***Hsf*** **genes in six Rosaceae species**

**Hsfs**	**Chinese white pear(29)**	**Apple(25)**	**Peach(17)**	**Strawberry(16)**	**Chinese plum(17)**	**European pear(33)**
*HsfA1a*	*Pbr025227.1*	*MDP0000517644*	*ppa004782m*	*gene13904*	*Pm023178*	*PCP005520.1*
*b*	*Pbr041026.1*	*MDP0000156337*	*ppa004559m*	*gene10474*	*Pm011227*	*PCP027354.1*
*c*	*Pbr031411.1*	*MDP0000232623*				*PCP027124.1*
*d*		*MDP0000259645*				*PCP011761.1*
*HsfA2a*	*Pbr019856.1*	*MDP0000489886*	*ppa007300m*	*gene02705*	*Pm005519*	*PCP044449.1*
*b*		*MDP0000243895*				*PCP016141.1*
*c*						*PCP034937.1*
*HsfA3a*	*Pbr005496.1*	*MDP0000131346*	*ppa015602m*	*gene30146*	*Pm026236*	*PCP016675.1*
*b*	*Pbr016805.1*	*MDP0000606400*				*PCP026047.1*
*c*		*MDP0000174161*				
*HsfA4a*	*Pbr000538.1*	*MDP0000155849*	*ppa006534m*	*gene23802*	*Pm010169*	*PCP025026.1*
*b*	*Pbr016090.1*		*ppa015468m*	*gene15872*	*Pm013905*	*PCP026169.1*
*c*	*Pbr022463.1*					*PCP024177.1*
*d*	*Pbr005379.1*					*PCP015400.1*
*HsfA5a*	*Pbr016487.1*	*MDP0000301101*		*gene06570*	*Pm007815*	*PCP002437.1*
*b*		*MDP0000613011*				
*HsfA6a*	*Pbr036788.1*		*ppa1027143m*	*gene29004*	*Pm009237*	*PCP030606.1*
*b*	*Pbr014670.1*					*PCP018714.1*
*c*	*Pbr018847.1*					
*HsfA7a*	*Pbr009953.1*		*ppa010224m*	*gene20347*	*Pm020253*	*PCP019575.1*
*b*	*Pbr012908.1*					*PCP022776.1*
*HsfA8a*	*Pbr012136.1*	*MDP0000191541*	*ppa006514m*		*Pm005887*	*PCP006787.1*
*b*		*MDP0000172376*				*PCP031284.1*
*HsfA9a*	*Pbr041474.1*	*MDP0000194672*	*ppa016533m*	*gene12667*	*Pm027197*	*PCP005035.1*
*b*	*Pbr015630.1*	*MDP0000319456*				*PCP027517.1*
*HsfB1a*	*Pbr025141.1*	*MDP0000527802*	*ppa009274m*	*gene24036*	*Pm026366*	*PCP024136.1*
*b*	*Pbr030422.1*	*MDP0000578396*				*PCP030007.1*
*HsfB2a*	*Pbr013953.1*	*MDP0000155667*	*ppa009180m*	*gene13301*	*Pm019357*	*PCP030684.1*
*b*			*ppa008441m*	*gene32416*	*Pm023788*	*PCP033244.1*
*c*						*PCP007662.1*
*HsfB3a*	*Pbr002020.1*	*MDP0000622590*	*ppa014675m*	*gene02464*		*PCP029678.1*
*b*	*Pbr030436.1*	*MDP0000202716*				*PCP024839.1*
*c*	*Pbr002038.1*					
*HsfB4a*	*Pbr019653.1*	*MDP0000209135*	*ppa026635m*		*Pm005297*	
*b*		*MDP0000129357*				
*HsfB5a*	*Pbr016270.1*		*ppa011804m*	*gene02408*	*Pm010031*	*PCP044895.1*
*b*						*PCP016888.1*
*HsfC1a*	*Pbr014107.1*	*MDP0000230456*	*ppa008830m*	*gene30881*	*Pm027421*	*PCP000545.1*
*b*	*Pbr016948.1*	*MDP0000320827*				*PCP022060.1*

### Gene features of *Hsf* genes

Gene features such as structural complexity and GC3 content have intense impacts on gene retention and evolution after WGD [36]. Hence, we investigated the features of *Hsf* genes in the Rosaceae, including gene length, intron length, GC content, and GC3 content (Additional file 3). The average GC and GC3 contents of the *Hsf* gene family were higher than the average levels for the whole genome in most of the six Rosaceae species. Additionally, the average intron lengths of these genes in each of the Rosaceae genomes, except that of European pear, were shorter than those at the whole genome level. Especially in peach and Chinese plum, the average gene lengths and intron lengths of *Hsf* genes were significantly shorter than the whole genome averages. These results may be related to the intron losses that occurred during the expansion and divergence of the *Hsf* gene family [37].

Furthermore, the exon–intron structures of the *Hsf* genes in Chinese white pear and the other Rosaceae species were resolved (Additional files 4 and 5). The structures of the genes in the different subfamilies were extremely similar; this observation further verified the precision of the classification. However, the location and number of introns and exons varied among the *Hsf* genes. Most members of the *Hsf* gene family in the Rosaceae contained one intron. Strikingly, *Hsf* genes comprised of multiple introns were found in all six Rosaceae species and were especially prevalent in apple, strawberry, and European pear (Additional file 5). Notably, *PcHsfA6b* contained 13 introns; this gene was extremely different from the other *Hsf* genes because of its large size (16595 bp) and the presence of TIFY and CCT_2 domains.

### Conserved protein domains in PbHsfs

Prediction of the typical signature domains of the PbHsfs protein sequences was conducted by comparing the identified PbHsfs with their well-characterized homologs from tomato, Arabidopsis, and apple [13,24,25,32]. Five conserved domains were identified by sequence alignment, and their positions in the protein sequences were determined (Table 3). All of the PbHsfs protein sequences contained the highly conserved DBD domain, consisting of a three helical bundle (H1, H2, and H3) and a four-stranded antiparallel β-sheet, in the N-terminal region. However, the length of the DBD domain was quite variable within the Hsf family. The presence of the coiled-coil structure characteristic of leucine zipper–type protein interaction domains, which is a property of the HR-A/B region, was instead predicted in all PbHsfs protein sequences using the MARCOIL tool. Furthermore, the majority of the PbHsfs protein sequences contained NES and NLS domains, which are essential for shuttling Hsfs between the nucleus and cytoplasm [13]. Additional sequence comparison identified AHA domains in the center of the C-terminal activation domains, as was expected in the A-type PbHsfs. By contrast, these domains were not identified in the B- and C-type PbHsfs.

**Table 3:** **Functional domains of PbHsfs**

**Gene name**	**DBD**	**HR-A/B**	**NLS**	**NES**	**AHA**
*PbHsfA1a*	16-109	126-196	(222) NKKRRLPR	(486) MNYITEQMQ	AHA(439) DIFWEQFLTAS
*PbHsfA1b*	16-109	126-196	(222) NKKRRLPR	(486) MNYITEQMQ	AHA(439) DIFWEQFLTAS
*PbHsfA1c*	39-132	153-220	(243) NKKRRLKQ	(499) MDNLTEKMG	AHA(452) DIEAFLKDWDD
*PbHsfA2a*	38-131	145-212	(227) KNR-X6-RKRR	(365) LVDQMGYL	AHA1(315) ETIWEELWSD
					AHA2(355) DWGEDLQD
*PbHsfA3a*	99-216	237-296	(308) KT-X10-RRKFVK	nd	AHA1(472) EDIWSMGFGV
					AHA2(491) ELWGNPVNY
					AHA3(511) LDVWDIGPLQ
					AHA4(527) IDKSPAHDS
					AHA1(471) EDIWSMDFDI
*PbHsfA3b*	103-215	237-295	(311) KDRGSSRVRRKFVK	nd	AHA2(489) NELLGNPVNY
					AHA3(510) LDVWDIDPLQ
					AHA4(526) INKWPAHES
*PbHsfA4a*	11-94	113-190	(208) RKRRLPR	(407) LTEQMGHL	AHA1(252) LTFWEDTIHD
					AHA2(356) DGFWEQFLTE
*PbHsfA4b*	11-94	113-188	(206) RKRRLPR	(410) LTEQMGHL	AHA1(250) LTFWEDTIHD
					AHA2(359) DVFWEQFLTE
*PbHsfA4c*	12-106	137-186	(204) KKRR	(429) FTNQIGRL	AHA1(252) LNFWEDFVHGI
					AHA2(378) DVFWEQCLTE
*PbHsfA4d*	12-106	137-187	(205) KKRR	(425) FRNQIGRP	AHA1(247) LNFWEDFLHGV
					AHA2(372) DVFWEQCLTE
*PbHsfA5a*	12-105	116-183	(194) RK-X_10_-KKRR	(477) AETLTL	AHA (431) DVFWEQFLTE
*PbHsfA6a*	31-125	154-204	(229) KKRRR	(344) LIEELGFL	AHA(312) DKGFWQDLFNE
					(271) EVSELNQFAM
*PbHsfA6b*	21-115	144-194	(210) RKELEKAVTKKRRR	(334) LIEELGFL	AHA(302) DKGYWQELFNE
*PbHsfA6c*	21-115	144-194	(210) RKELEKAVTKKRRR	(334) LIEELGFL	AHA(302) DKGYWQDLFNE
*PbHsfA7a*	44-138	167-217	(232) KKKELEEAMTKKRRR	(351) LADRLGYV	AHA(319) DEGFWEELFSE
*PbHsfA7b*	44-138	167-217	(232) KKKELEEAMTKKRRR	(344) LADRLGYF	AHA(312) DEGFWEELLSE
*PbHsfA8a*	18-111	129-198	(177) RNRLR	(389) TEQMGHL	AHA (308) DGAWEQFLLA
*PbHsfA9a*	95-182	247-315	(268) KR-X12-KRRR	(401) FYQELEDL	AHA(467) PCDWSAYVSHS
*PbHsfA9b*	139-239	241-308	(324) KR-X8-KRRR	(258) LKKDQD	AHA(460) PCDWSAYVSNS
*PbHsfB1a*	6-99	142-191	(246) KGDEKMKGKK	nd	nd
*PbHsfB1b*	66-99	42-191	(246) KGEEKMKGKK	(223) LDMEGG	nd
*PbHsfB2a*	42-135	174-217	(187) RLRK	nd	nd
*PbHsfB3a*	19-112	149-194	(223) RKRKR	(208) PKLFGVRLE	nd
*PbHsfB3b*	19-112	149-194	(223) RKRKR	(208) PKLFGVRLE	nd
*PbHsfB3c*	22-116	149-194	(180) KRKCK (223) RKRKR	(208) LKLFGVRLE	nd
*PbHsfB4a*	21-114	179-239	(326) KNTK-X_9_-KKR	(367) LEKDDLGLHLM	nd
*PbHsfB5a*	11-111	151-188	nd	(151) LRKQKLELQV	nd
*PbHsfC1a*	9-102	121-173	(197) KKRR	nd	nd
*PbHsfC1b*	9-102	126-178	(225) KKRR	nd	nd

nd: no motifs detectable by sequence similarity search.

**Figure 4:**
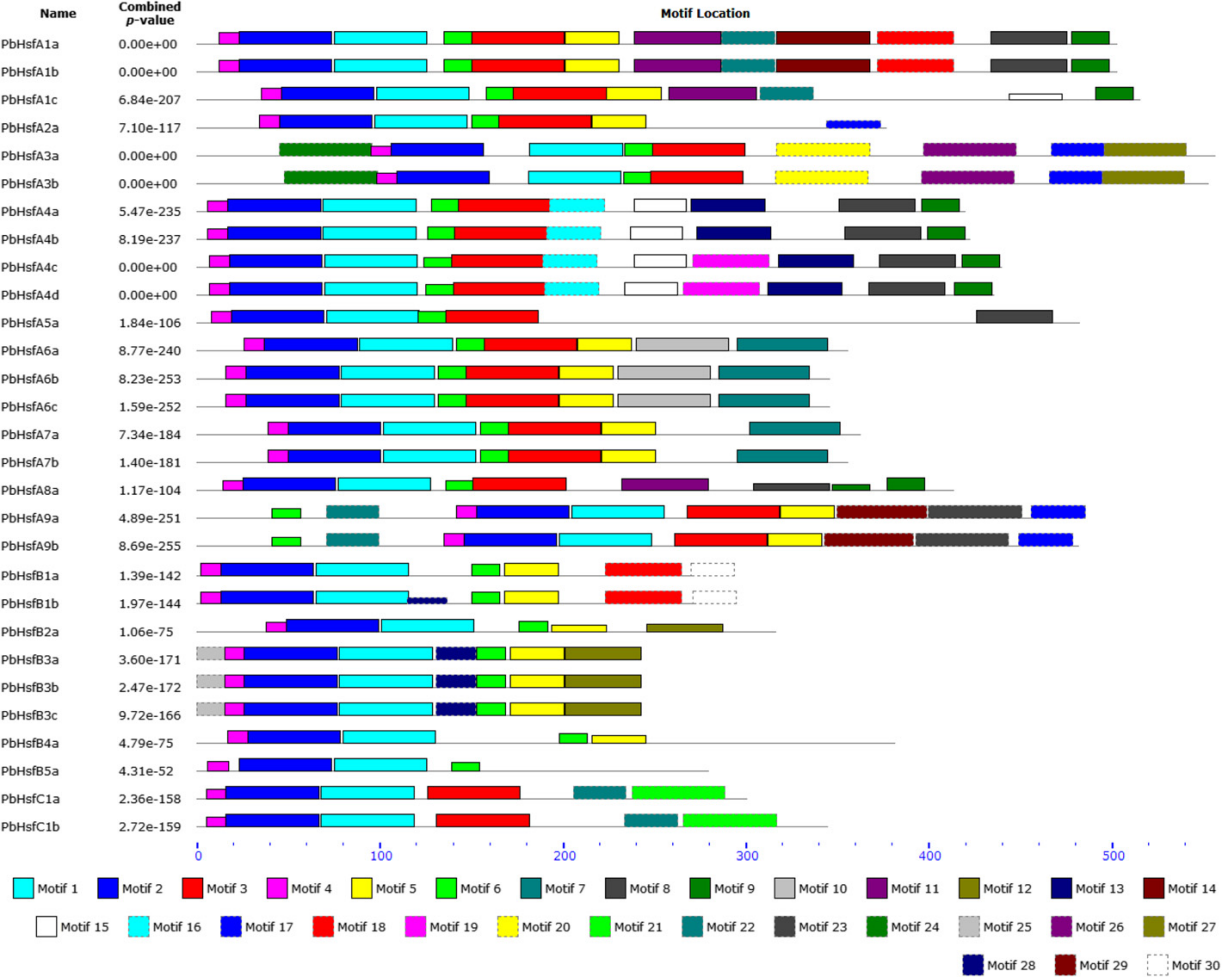
**Motifs identified by MEME tools in Chinese white pear Hsfs.** Thirty motifs (1 to 30) were identified and indicated by different colors. Motif location and combined *p*-value were showed.

The Multiple EM for Motif Elicitation (MEME) motif search tool was used to predict and verify domains in the PbHsf protein sequences. Thirty corresponding consensus motifs were detected (Figure 4; Additional file 6). The number of motifs contained in the PbHsf protein sequences was quite variable. The members of class A contained the most conserved motifs, with the largest number (12) detected in PbHsfA1a and PbHsfA1b. Class C members possessed the fewest motifs, while class B PbHsfs contained an intermediate number. Regarding the DBD domain, motifs 1, 2, and 4 were found in 29 members of the PbHsfs family. The coiled-coil structure motifs 3, 5, 6 were detected in all members of the PbHsfs family. All class B proteins exhibited the coiled-coil region motifs 5 or 6, whereas motifs 3 and 6 were detected in classes A and C. The conserved motifs 3, 5, 6, 12, 16, 18, and 20 were identified as NLS. Motifs 3, 5, 16, and 20 were representative NLS domains in class A, while NLS domains were represented by motifs 6, 12, and 18 in class B. Furthermore, motifs 9, 12, 17, 18, and 23 represented NES domains; motifs 9, 17, and 23 were only observed in class A, while motifs 12 and 18 were seen only in class B. Motifs 7, 8, 10, 15, 17, and 27 was identified as characteristic AHA domains. Despite the variability in size and sequence, predicted DBD domain, HR-A/B domain and NLS domain were observed in each PbHsfs through the combination of the two methods.

### Synteny analyses reveal the origin and expansion of the *Hsf* gene family

Several gene duplication modes drive the evolution of protein-coding gene families, including WGD or segmental duplication, tandem duplication, and rearrangements at the gene and chromosomal level [38]. We detected the origins of duplicate genes for the *Hsf* genes family in five Rosaceae genomes using the MCScanX package. Each member of *Hsf* gene family was assigned to one of five different categories: singleton, WGD, tandem, proximal or dispersed. Different patterns of gene duplication contributed differentially to the expansion of the *Hsf* gene family in the investigated taxa (Table 4). Remarkably, 75.9% (22) of the *Hsf* genes in Chinese white pear and 68% (17) of those in apple were duplicated and retained from WGD events, compared to only 35.3% (6) in peach, 25% (4) in strawberry, and 23.5% (4) in Chinese plum. The recent lineage-specific WGD events (30–45 MYA) in pear and apple likely resulted in the higher proportions of WGD-type *Hsf* gene duplications observed in these species. However, the proportions of dispersed *Hsf* gene duplication in peach (64.7%), strawberry (75%), and Chinese plum (76.5%) were considerably higher than in pear (17.2%) and apple (20%). Peach, strawberry, and Chinese plum have not experienced a WGD since their divergence from apple and pear. Therefore, genome rearrangements, gene losses, and RNA- and DNA-based transposed gene duplications may account for the larger proportions of dispersed duplicates in these species. These results showed that WGD or segmental duplication and dispersed gene duplication played critical roles in the expansion of the *Hsf* gene family in the Rosaceae.

**Table 4:** **Numbers of** ***Hsf*** **genes from different origins in five Roseceae genomes**

**Species**	**No. of** ***Hsf*** **genes**	**No. of** ***Hsf*** **genes from different origins (percentage)**				
		**Singleton**	**WGD**	**Tandem**	**Proximal**	**Dispersed**
Chinese white pear	29	0	22(75.9)	0	2(6.9)	5(17.2)
Apple	25	0	17(68.0)	0	3(12.0)	5(20.0)
Peach	17	0	6(35.3)	0	0	11(64.7)
Strawberry	16	0	4(25)	0	0	12(75)
Chinese plum	17	0	4(23.5)	0	0	13(76.5)

Collinearity and synteny are traditionally identified by looking for both intra- and intergenomic pairwise conservation blocks. To further investigate the potential evolutionary mechanisms of the *PbHsf* gene family, we performed all-vs.-all local BLASTP to identify synteny blocks, using a method similar to that used for the Plant Genome Duplication Database (PGDD), across the entire Chinese white pear genome. The dates of segmental duplications can be inferred through this method; if two or more syntenic regions exist in one species, these regions are considered to be segmental duplications.

**Figure 5:**
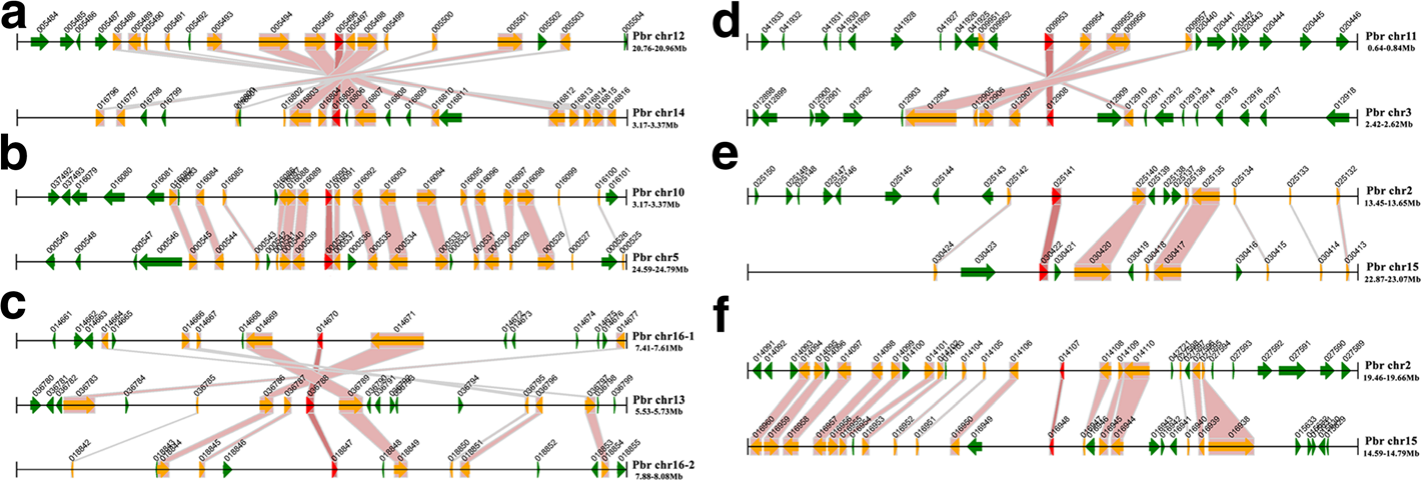
**Segmental duplication between members of the** ***Hsf*** **family in Chinese white pear. (a)** *PbHsfA3a*(Pbr005496) and *PbHsfA3b*(Pbr016805), **(b)** *PbHsfA4a*(Pbr000538) and *PbHsfA4b*(Pbr016090), **(c)** *PbHsfA6a*(Pbr036788) and *PbHsfA6b*(Pbr014670) and *PbHsfA6a*(Pbr036788) and *PbHsfA6c*(Pbr018847), **(d)** *PbHsfA7a*(Pbr009953) and *PbHsfA7b*(Pbr012908), **(e)** *PbHsfB1a*(Pbr025141) and *PbHsfB1c*(Pbr030422), **(f)** *PbHsfC1a*(Pbr014107) and *PbHsfC1b*(Pbr016948). The figure shows a region of 100 kb on each side flanking the *Hsf* genes. Homologous gene pairs are connected with bands. The chromosome segment is indicated by black horizontal line, and the broad line with arrowhead represents gene and its transcriptional orientation. The text besides the gene is the gene locus identifier suffix. The *Hsf* genes are shown in red, homologous genes are shown in yellow, and other genes shown in green.

Conserved synteny was observed in 22 regions containing *Hsf* genes across the Chinese white pear genome (Figure 5), and these syntenic blocks included most of the *Hsf* genes (Table 5). We observed strongly conserved synteny in some of these blocks, several of which contained over 100 syntenic gene pairs (data not shown). These results support the occurrence of chromosome segment duplication or WGD in Chinese white pear [34]. A total of 13 segmentally duplicated gene pairs were found in the *PbHsf* gene family. Chromosomes 4 and 7 were not involved in any duplication events.

**Table 5:** **Synteny analysis of** ***Hsf*** **gene regions in Chinese white pear genome**

**Duplicated** ***Hsf*** **gene 1**	**Duplicated** ***Hsf*** **gene 2**	**Mean Ks**	**Homologous gene pairs in 200 kb**	**Genes in 200 kb**
*PbHsfA2a*	*PbHsfA9a*	2.13	6	30
*PbHsfA2a*	*PbHsfA9b*	1.60	2	30
*PbHsfA3a*	*PbHsfA3b*	0.25	14	21
*PbHsfA4a*	*PbHsfA4b*	0.25	17	25
*PbHsfA4a*	*PbHsfA4d*	2.35	1	12
*PbHsfA4c*	*PbHsfA4d*	0.31	5	12
*PbHsfA6a*	*PbHsfA6b*	0.21	7	17
*PbHsfA6a*	*PbHsfA6c*	0.20	8	14
*PbHsfA7a*	*PbHsfA7b*	0.24	6	20
*PbHsfA7b*	*PbHsfA6b*	1.51	2	17
*PbHsfA7b*	*PbHsfA6c*	1.79	2	14
*PbHsfB1a*	*PbHsfB1b*	0.32	8	12
*PbHsfC1a*	*PbHsfC1b*	0.24	17	28

We chose six consecutive homologous gene pairs on each side flanking the *Hsf* genes to calculate the mean Ks, and calculated the number of genes in 200 kb according to the segment with less genes in 200 kb.

### Ks value and Ka/Ks ratio reveal dates and driving forces of evolution

The Ks value (synonymous substitutions per site) is widely used to estimate the evolutionary dates of WGD or segmental duplication events. Based on Ks values, two genome-wide duplication events were observed in the apple genome: the paleoduplication event corresponding to the γ triplication (Ks ~1.6) and a recent WGD (Ks ~0.2) [39]. Similarly to that in apple, the ancient WGD (Ks ~1.5–1.8) in pear resulted from a paleohexaploidization (γ) event that took place ~140 MYA [40], while the recent WGD (Ks ~0.15–0.3) in pear was inferred to have occurred at 30–45 MYA [34,39]. All members of the rosid clade have undergone paleohexaploidization (γ) [39,41–43]. Therefore, we used Ks values to estimate the evolutionary dates of the segmental duplication events among the *PbHsf* gene family. The mean Ks of the *Hsf* duplicated gene pairs in the syntenic region are shown in Table 5. The Ks values for the *PbHsf* gene pairs ranged from 0.20 to 2.35. We further inferred that the segmental duplications *PbHsfA2a* and *PbHsfA9b* (Ks ~1.60), *PbHsfA7b* and *PbHsfA6b* (Ks ~1.51), and *PbHsfA7b* and *PbHsfA6c* (Ks ~1.79) may have arisen from the γ triplication (~140 MYA). Furthermore, many duplicated gene pairs had similar Ks values (0.21–0.32), suggesting that these duplications may have been derived from the same recent WGD (30~45 MYA). Surprisingly, two duplicated gene pairs (*PbHsfA2a* and *PbHsfA9a*, *PbHsfA4a* and *PbHsfA4d*) possessed higher Ks values (2.13-2.15), suggesting that they might have stemmed from a more ancient duplication event.

The determination of orthology is an essential part of comparative genomics. Identification of orthology using synteny analysis has been employed in many studies [44–46]. According to the identified synteny relationships, we identified orthologous pairs of *Hsf* genes among five Rosaceae species (Table 6 and Additional file 7). A total of 29 *PbHsf* genes were found in orthologous blocks within five Rosaceae species, while 18 in apple, 17 in peach, 15 in strawberry, and 16 in Chinese plum. The numbers of orthologous pairs between Chinese white pear and other four Rosaceae species (apple, peach, strawberry and Chinese plum) are 30, 32, 26 and 29, respectively. The average Ks values of the *Hsf* orthologs between Chinese white pear and apple, peach, strawberry, or Chinese plum ranged from 0.21 to 0.75 (Additional file 8). The *Hsf* orthologs between Chinese white pear and apple possessed the lowest average Ks value (0.21), suggesting that the evolutionary distance was closest between these species. The average Ks values of the *Hsf* orthologs between Chinese white pear and peach, Chinese plum, and strawberry were 0.55, 0.53, and 0.75, respectively.

**Table 6:** **The orthology of** ***Hsf*** **genes in five Rosaceae species**

	**Chinese pear**	**Apple**	**Peach**	**Strawberry**	**Chinese plum**
*HsfA1*	*PbHsfA1a*	*MdHsfA1b*	*PpHsfA1b*	*FvHsfA1b*	*PmHsfA1b*
	*PbHsfA1b*	*MdHsfA1b*	*PpHsfA1b*	*FvHsfA1b*	*PmHsfA1b*
	*PbHsfA1c*		*PpHsfA1a*		*PmHsfA1a*
*HsfA2*	*PbHsfA2a*	*MdHsfA2a,2b,9b*	*PpHsfA2a,9a*	*FvHsfA2a,9a*	*PmHsfA2a,9a*
*HsfA3*	*PbHsfA3a*	*MdHsfA3a,b*	*PpHsfA3a*	*FvHsfA3a*	*PmHsfA3a*
	*PbHsfA3b*	*MdHsfA3a,b*	*PpHsfA3a*	*FvHsfA3a*	*PmHsfA3a*
*HsfA4*	*PbHsfA4a*		*PpHsfA4a*	*FvHsfA4a,b*	*PmHsfA4a*
	*PbHsfA4b*		*PpHsfA4a*	*FvHsfA4a,b*	*PmHsfA4a*
	*PbHsfA4c*		*PpHsfA4b*	*FvHsfA4b*	*PmHsfA4b*
	*PbHsfA4d*		*PpHsfA4a,4b*	*FvHsfA4b*	*PmHsfA4b*
*HsfA5*	*PbHsfA5a*	*MdHsfA5a*		*FvHsfA5a*	*PmHsfA5a*
*HsfA6*	*PbHsfA6a*		*PpHsfA6a*	*FvHsfA6a*	*PmHsfA6a*
	*PbHsfA6b*		*PpHsfA6a,7a*	*FvHsfA6a,7a*	*PmHsfA6a,7a*
	*PbHsfA6c*		*PpHsfA6a,7a*	*FvHsfA6a,7a*	*PmHsfA6a,7a*
*HsfA7*	*PbHsfA7a*		*PpHsfA7a*		*PmHsfA7a*
	*PbHsfA7b*		*PpHsfA6a,7a*	*FvHsfA7a*	*PmHsfA6a,7a*
*HsfA8*	*PbHsfA8a*	*MdHsfA8a,8b*	*PpHsfA8a*		*PmHsfA8a*
*HsfA9*	*PbHsfA9a*	*MdHsfA9a,9b*	*PpHsfA9a*	*FvHsfA9a*	*PmHsfA9a*
	*PbHsfA9b*	*MdHsfA9a,9b*	*PpHsfA9a*	*FvHsfA9a*	*PmHsfA9a*
*HsfB1*	*PbHsfB1a*	*MdHsfB1a*	*PpHsfB1a*	*FvHsfB1a*	*PmHsfB1a*
	*PbHsfB1b*	*MdHsfB1a*	*PpHsfB1a*	*FvHsfB1a*	
*HsfB2*	*PbHsfB2a*	*MdHsfB2a*	*PpHsfB2a,2b*	*FvHsfB2a,2b*	*PmHsfB2a*
*HsfB3*	*PbHsfB3a*	*MdHsfB3a*	*PpHsfB3a*		
	*PbHsfB3b*	*MdHsfB3a,3b*	*PpHsfB3a*		
	*PbHsfB3c*	*MdHsfB3a,3b*	*PpHsfB3a*		
*HsfB4*	*PbHsfB4a*	*MdHsfB4a,4b*	*PpHsfB4a*		*PmHsfB4a*
*HsfB5*	*PbHsfB5a*		*PpHsfB5a*	*FvHsfB5a*	*PmHsfB5a*
*HsfC1*	*PbHsfC1a*	*MdHsfC1a,1b*	*PpHsfC1a*	*FvHsfC1a*	*PmHsfC1a*
	*PbHsfC1b*	*MdHsfC1a,1b*	*PpHsfC1a*	*FvHsfC1a*	*PmHsfC1a*

Genes in the same row are putative orthologs within five species. Note that one *PbHsf* gene may anchor to multiple *Hsf* genes in another Rosaceae species, each of those *Hsf* genes was identified as the ortholog for this *PbHsf* gene.

Negative selection (purifying selection) is the process by which deleterious mutations are removed. Conversely, positive selection (Darwinian selection) accumulates new advantageous mutations and spreads them through the population [47]. To further detect which selection process drove the evolution of the *Hsf* gene family, we also analyzed the Ka value (nonsynonymous substitutions per site), Ka/Ks ratio of paralogs in the Rosaceae *Hsf* gene family using coding sequences (CDS) (Additional file 9). The Ka/Ks ratio measures the direction and magnitude of selection: a value greater than one indicates positive selection, a value of one indicates neutral evolution, and a value less than one indicates purifying selection [48]. All Ka/Ks ratios of the paralogous genes were less than one, implying that purifying selection was the primary influence on the *Hsf* family genes.

### Expression of *Hsf* family genes in pear fruit

The expression of *PbHsf* genes was investigated at the transcriptional level. At first, the Chinese white pear expressed sequence tags (ESTs) database was searched for the *Hsf* genes to verify the accuracy of the previous genomic predictions. These results provided reliable transcriptional evidence for most of these *PbHsf* genes (Additional file 10). Of the 29 predicted *PbHsf* genes, 22 were found to have EST hits with highest score. A total of 44 EST hits were found for all *PbHsf* genes, with the greatest number (four each) for *PbHsfA1a* and *PbHsfB2a.* These results provide credible support for the identification of *PbHsf* family genes. However, no EST hits were identified for *PbHsfA6a*, *PbHsfA6b*, *PbHsfA6c*, *PbHsfA9b*, *PbHsfB3a*, *PbHsfB3b*, and *PbHsfB5a* against the EST database. The functional roles of these genes will require further investigation.

To further explore the expression patterns of *Hsf* family genes in Chinese white pear, transcriptome sequencing analysis was conducted using fruit samples harvested from pear trees under field conditions and naturally increased temperatures. We took fruit samples from spring to summer 2012 at four different developmental stages (S1-S4) corresponding to different temperature ranges. The first sampling, used as a reference, was conducted on April 22 (S1) at 26°C/15°C (day/night; max/min), corresponding to 15 days after flowering (DAF). Subsequent samples were taken on May 13 at 26°C/19°C (S2, 36 DAF), June 27 at 27°C/21°C (S3, 80 DAF), and July 28 at of 36°C/28°C (S4, 110 DAF).

**Figure 6:**
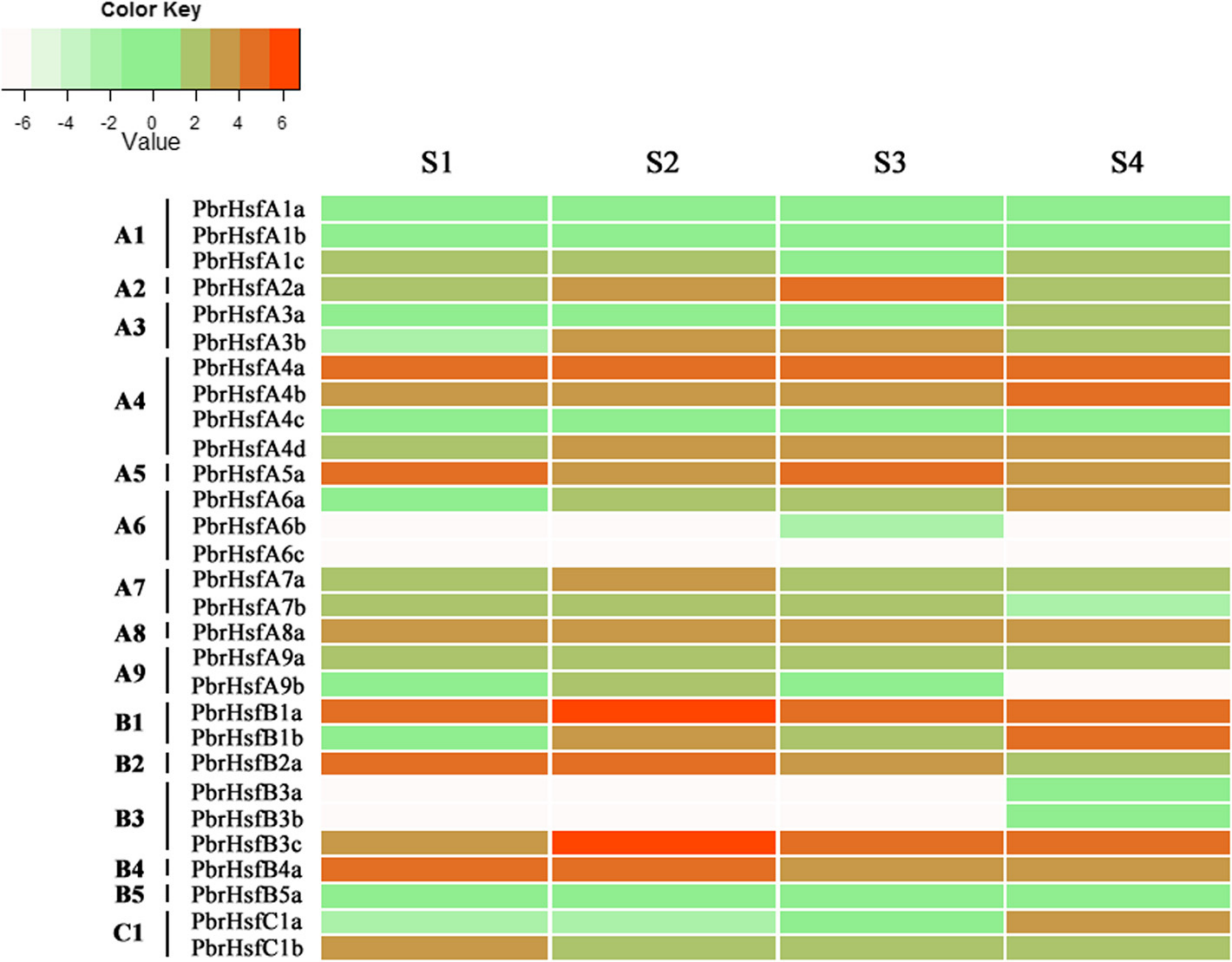
**Heatmap of expression level of** ***Hsf*** **genes in Chinese white pear fruit.** Transcriptome data were used to measure the expression level of *Hsf* genes. The groups A1-C1 on the left correspond to different subfamilies. S1-S4 correspond to four different developmental stages: on 22nd April (S1), 13rd May (S2), 27th June (S3), and 28th July (S4). Color scale at the top represents log2 transformed RPKM (reads per kilobase per million) values. Light green indicates low expression and red indicates high expression. Heatmap was generated using R.

The results of transcriptome sequencing analysis are shown in Figure 6 (Additional file 11), and the *PbHsf* genes were responsive to the increased temperatures. RPKM (reads per kilobase per million) values were used to measure the expression level of the *PbHsf* genes. The expression patterns of the 29 *PbHsf* genes were very diverse, and most *PbHsf* genes exhibited some degree of stage specificity. Only *PbHsfA6c* exhibited no expression. Twenty-four genes were detected across the four fruit developmental stages. Five genes (*PbHsfA4a*, *PbHsfA5a*, *PbHsfA8a*, *PbHsfB1a*, and *PbHsfB3c*) presented high expression in all four stages. Moreover, six *PbHsf* genes (*PbHsfA3a*, *PbHsfA4b*, *PbHsfA4d*, *PbHsfA6a*, *PbHsfB1b*, and *PbHsfC1a*) showed increasing transcript expression with rising temperature, while *PbHsfA9a*, *PbHsfA9b*, and *PbHsfB4a* expression decreased with the increased temperatures. However, *PbHsfB3a* and *PbHsfB3b* showed only relatively little expression in stage S4, and *PbHsfA6b* was expressed only in S3. Additionally, the transcriptional changes of *PbHsfA1a*, *PbHsfA1b*, and *PbHsfA1c* were not obviously associated with temperature.

## Discussion

Members of the *Hsf* gene family have been identified and analyzed in different land plant species [13]. The number and composition of *Hsf* family members differ in various plants. Ancient polyploidy events (also known as WGDs) and additional recent lineage-specific WGDs have presumably resulted in varying numbers of *Hsf* genes within flowering plants. In this study, the sizes of the *Hsf* gene families identified from the six Rosaceae genomes are diverse. The number of *Hsf* genes in Chinese white pear (29), European pear (33), and apple (25) are greater than those in peach (17), strawberry (16), and Chinese plum (17). Pear and apple were inferred to have undergone a recent lineage-specific WGD, while peach, strawberry, and Chinese plum did not experience this event [49]. Therefore, this recent WGD event likely led to the different numbers of *Hsf* genes in the investigated Rosaceae species.

Different patterns of gene duplication, such as genome-wide, tandem, and dispersed duplications, contribute differentially to the expansion of specific gene families in plant genomes [50–52]. Some large gene families, including the APETALA 2/ethylene-responsive element binding factor (AP2/ERF) and WRKY, are more likely to expand by segmental and tandem duplications [53,54]. Conversely, gene families such as MADS (MINICHROMOSOME MAINTENANCE1, AGAMOUS, DEFICIENS and SERUM RESPONSE FACTOR)-box, and NBS-LRR (nucleotide-binding site-leucine-rich repeat) expand primarily through transposed duplications [50]. It has been estimated that more than 90% of the increase in regulatory genes in the *Arabidopsis* lineage has been caused by genome duplications [55]. Recent genome-wide studies have revealed that the pear and apple genomes experienced at least two genome duplications, one ancient and one before the pear-apple divergence [34]. Indeed, in this study, the results of the synteny analysis verified that the expansion of the *Hsf* gene family in Chinese white pear and apple was derived primarily from WGD or segmental duplications. This situation, in which segmental *Hsf* gene duplications were more frequent than tandem duplications, also occurred in *Arabidopsis*, maize, and poplar [32,33,56]. However, dispersed duplications were the major drivers of *Hsf* gene family expansion in peach, strawberry, and Chinese plum. The genomes of these three species have not experienced recent WGD. The genome rearrangements, gene losses, and gene transposition and retrotransposition after the ancient polyploidy event may have had a comparatively stronger impact on the evolution of the *Hsf* gene family in peach, strawberry, and Chinese plum.

Polyploidy through WGD is often followed by rapid gene loss, and genome rearrangements have been widely recognized as important in the evolution of plant genomes [57]. The retention of genes duplicated through WGD is biased in plant genomes and has been shown to be nonrandom across gene families [36,50]. For example, in *Arabidopsis*, genes encoding transcription factors, protein kinases, and ribosomal proteins have been preferentially retained after WGD [55,58,59]. In recent years, several models have been applied to elucidate the evolutionary fates and biased retention of duplicated genes, such as subfunctionalization, neofunctionalization, and dosage balance [60]. Recent studies have strongly supported the hypothesis that the overretention of duplicated genes derived from WGD is intensely correlated with greater structural complexity, highly conserved domains, lower evolutionary rates, and higher GC3 content in the plant genome, suggesting that multiple models may together drive the evolution of genes duplicated after WGDs [36]. Our present study showed that the *Hsf* gene family has undergone specific expansion and been preferentially retained. Rosaceae *Hsf* family genes possess shorter intron lengths and higher GC and GC3 contents than the genome average, contain several highly conserved functional domain, and present lower ka/ks ratios, corresponding to a slower evolutionary rate. These results were consistent with previously obtained results [36], implying that *Hsf* genes have been functionally stable over recent years and may serve as good targets for dosage balance selection [50].

Pear and apple belong to the Maloideae, peach and Chinese plum belong to the Prunoideae, and strawberry belongs to the Rosoideae. The divergence of the Rosoideae occurred prior to that of the Maloideae and Prunoideae. Therefore, the Maloideae and Prunoideae have a closer evolutionary relationship. Phylogenetic analysis of the *Hsf* genes in the six Rosaceae species showed that *PbHsfs*, *MdHsfs*, and *PcHsfs* were clustered together in the phylogenetic tree, while *PpHsfs* and *PmHsfs* had a closer relationship, as was consistent with the evolutionary history among the three subfamilies. These observations suggest that the expansion of these *Hsf* genes occurred before the divergence of the Rosaceae species. Furthermore, the majority of the *PbHsf* genes were related more closely to *PtHsfs* than to *AtHsfs*. This result may be explained by the fact that both *Pyrus* and *Populus* belong to the Fabids clade [61] and are both trees subjected to prolonged environmental stress. All three *Hsf* classes (A, B, and C) identified in Populus, *Arabidopsis*, and pear imply that the *Hsf* genes originated prior to the divergence of the three species. Additionally, *Hsf* members of the three classes have been detected in different lineages of monocots and dicots. In light of the present results, we inferred that the expansion of the *Hsf* gene family may have occurred in the common ancestor of angiosperms.

The functional diversification of *Hsf* genes has been observed in several plant species. *HsfA1a* has been reported as a single master regulator gene in tomato [22]. *AtHsfA1a* and *AtHsfA1b* are known to be involved in the early response to heat stress (HS) in Arabidopsis [62,63]. *AtHsfA2* enhances and maintains the HS response when plants are subjected to long-term or repeated cycles of HS [64,65]. Previous data regarding *Hsf* expression in apple trees exposed to naturally increased temperatures are also available. For example, the A1-type *MdHsf* genes are expressed at the same level regardless of temperature in apple leaves, while *MdHsfA2a-b*, *MdHsfA3b-c* are strongly induced by high temperature [32]. Similarly to those of *MdHsf* genes, the transcriptional expression levels of A1-type *PbHsf* genes showed no significant changes as plants were exposed to naturally increasing temperatures. *PbHsfA4a*, *PbHsfA5a*, and *PbHsfA8a* were all strongly induced across the four stages of fruit development, indicating that the subclasses *PbHsfA4*, *PbHsfA5*, and *PbHsfA8* were closely related with maintaining the heat shock response of pear trees subjected to high-temperature conditions. *PbHsfA3a*, *PbHsfA4b*, *PbHsfA4d*, and *PbHsfA6a* were upregulated under naturally increased temperatures, implying that these genes play a critical role during heat stress response.

The members of the B class *Hsf* genes may act as transcription repressors or coactivators regulating acquired thermotolerance during HS regimes [66–68]. The function of class C *Hsf* genes has not yet been fully identified. Notably, *PbHsfB1a* and *PbHsfB3c* were highly expressed in all four of the studied stages (S1, S2, S3, S4). *PbHsfB1b* and *PbHsfC1a* were upregulated under rising temperature, suggesting that these genes may play important roles in the response to high temperatures in pear. However, further investigations will be required to determine the functions of class B and C *Hsf* genes in pear. Some *PbHsf* genes showed unaltered or downregulated expression under increased temperatures, suggesting that these genes may operate at other signal transduction pathways in the complex regulatory network of plant stress response [69,70]. We also compared the expression levels of 13 duplicated gene pairs in pear *Hsf* gene family; differences were detected between the two members of each gene pair. This result suggested that the duplicated genes exhibited significant functional divergence regarding the response to heat stress.

## Conclusions

A total of 137 full-length *Hsf* genes were identified in the six Rosaceae genomes, and the Chinese white pear genome contained 29 *Hsf* genes. According to the structural characteristics of the proteins, phylogenetic analysis, and comparison with homologues from Populus and *Arabidopsis*, the *Hsf* genes were grouped into three classes (A, B, and C). Collinearity analysis suggested that the recent WGD (30–45 MYA) may have driven the large scale expansion of the *Hsf* gene family in Chinese white pear and apple. Purifying selection is the major force acting upon *Hsf* family genes. EST and transcriptome sequencing analysis provided evidence of the identified *PbHsf* genes and revealed that they play an important role in heat stress response and fruit development. Considered together, these results constitute a foundation for further studies examining the functioning and complexity of the *Hsf* gene family in the Rosaceae.

## Methods

### Identification and classification of *Hsfs*

The Chinese white pear (*Pyrus bretschneideri*) genome sequence was downloaded from the pear genome project (http://peargenome.njau.edu.cn/) [34]. The genome sequences of apple, peach, and strawberry were downloaded from Phytozome (http://phytozome.jgi.doe.gov/pz/portal.html#), and the European pear genome sequence was download from the Genome Database for Rosaceae (GDR) (http://www.rosaceae.org/). The Chinese plum genome sequence was downloaded from the *Prunus mume* Genome Project (http://prunusmumegenome.bjfu.edu.cn/index.jsp). Initially, the Arabidopsis Hsf protein sequences At4g17750 (class A), At4g36990 (class B), and AT3g24520 (class C) downloaded from The Arabidopsis Information Resource (TAIR) [71] (http://www.arabidopsis.org/) were used as queries to perform BLAST against the six Rosaceae genome databases. Additionally, the seed alignment file for the Hsf domain (PF00447) obtained from the Pfam database [72] was used to build a HMM file using the HMMER3 software package [73]. HMM searches were then performed against the local protein databases of the six Rosaceae species using HMMER3. A total of 185 candidate *Hsf* genes were identified from the six Rosaceae species. Moreover, we checked the physical localizations of all candidate *Hsf* genes and rejected redundant sequences with the same chromosome location. Furthermore, all obtained Hsf protein sequences were again analyzed in the Pfam database to verify the presence of DBD domains. DBD domains and coiled-coil structures were also detected by the SMART and MARCOIL programs (SMART: http://smart.embl-heidelberg.de/, MARCOIL: http://toolkit.tuebingen.mpg.de/marcoil). Those protein sequences lacking the DBD domain or a coiled-coil structure were removed.

To identify signature domains, the PbHsf protein sequences were compared to the Hsf proteins of *Arabidopsis thaliana*, *Solanum lycopersicum, Populus trichocarpa*, and *Malus domestica* by amino acid sequence alignment using ClustalW2 (http://www.ebi.ac.uk/Tools/msa/clustalw2/). The protein sequences of those four species were downloaded from Heatster (http://www.cibiv.at/services/hsf/). PredictNLS [74] and NetNES 1.1 [75] were also used to predict NLS and NES domains, respectively. All full-length amino acid sequences of the PbHsfs were also used by the MEME tool [76] to identify conserved domain motifs. The parameters were set as follows: maximum numbers of different motifs, 30; minimum motif width, 6; maximum motif width, 50. Hsf names were assigned based on the original nomenclature established for the *Arabidopsis thaliana* Hsf family [13,24]. Classification of the three different groups A, B, and C was based on observations of the oligomerization domains [24].

### Chromosomal location and gene structure of *Hsfs*

The chromosomal location information of the *Hsf* genes was obtained from genome annotation documents. The data were then plotted using Circos software [77]. The gene structures of the *Hsf* genes were drawn using Gene Structure Display Server [78].

### Phylogenetic analysis

First, a neighbor joining phylogenetic tree was created using the full-length protein sequences of Hsf from six Rosaceae species. Second, another phylogenetic tree was constructed using the N-terminal Hsf protein sequences containing the DBD and HR-A/B regions and parts of the linker between these two regions from *Pyrus bretschneideri*, *Arabidopsis thaliana*, and *Populus trichocarpa* [24,33] using the NJ method in MEGA (version 6.0) [79]. NJ analysis was performed with the Poisson model. Bootstrap analysis was conducted with 1000 replicates to assess the statistical support for each node.

### Synteny analysis

The analysis of synteny among the six Rosaceae genomes was conducted locally using a method similar to that developed for the PGDD (http://chibba.agtec.uga.edu/duplication/) [80]. First, BLASTP was performed to search for potential homologous gene pairs (E < 1 e^−5^, top 5 matches) across multiple genomes. Then, these homologous pairs were used as the input for MCScanX to identify syntenic chains [81,82]. *MCScanX* was further used to identify WGD/segmental, tandem, proximal and dispersed duplications in the *Hsf* gene family.

### Calculating Ka and Ks of the *Hsf* gene family

MCScanX downstream analysis tools were used to annotate the Ka and Ks substitution rates of syntenic gene pairs. The mean Ks values of orthologous *Hsf* gene pairs between Chinese white pear and the other Rosaceae species were calculated using all homologous gene pairs located in the same synteny block. KaKs_Calculator 2.0 was used to determine Ka and Ks [83]. To date segmental duplication events, six consecutive homologous gene pairs on each side flanking the *Hsf* genes were chosen to calculate the mean Ks. For those segments with fewer than 12 homologous genes, all available anchor pairs were used [46].

### Expression analysis by ESTs

We conducted a local BLASTN against Chinese white pear EST libraries to find the corresponding record for each putative *PbHsf* genes using the following parameters: maximum identity > 95%, length > 200 bp, and E-value <10^−10^.

### Plant material and transcriptome sequencing

We conducted this experiment in 2012 on pear trees (cultivar ‘Dangshansuli’) planted in the experimental orchard of the College of Horticulture at Nanjing Agricultural University. Fruit samples were taken from homogeneous trees, and three biological replicates were collected. Pear fruit were harvested between April and July 2012 from trees grown under the natural variability of weather and climate. Total RNA was extracted for RNA sequencing, and RNA sequencing libraries were constructed using an Illumina standard mRNASeq Prep Kit (TruSeq RNA and DNA Sample Preparation Kits version 2). Transcriptome sequencing and assembly were performed on an Illumina Hi-seq 2000 Sequencer.

### Availability of supporting data

The data sets supporting the results of this article are included within the article and its additional files. The phylogenetic data including data matrices, phylogenetic trees, and analysis steps have been submitted to TreeBASE database under accession number 16806 (http://purl.org/phylo/treebase/phylows/study/TB2:S16806). The raw RNA-seq reads are available from the National Center for Biotechnology Information repository under accession PRJNA185970 (http://www.ncbi.nlm.nih.gov/bioproject/PRJNA185970). The EST datasets are available from the pear genome project (http://peargenome.njau.edu.cn/).

## Additional files

Additional file 1:
**Location of** ***Hsf*** **genes in strawberry and Chinese plum.** *Hsf* genes in strawberry(*FvHsf*), and Chinese plum (*PmHsf*) were mapped on the different chromosomes. Chromosome number is indicated on the inner side and highlighted red short lines in the inner circle correspond to different *Hsf* genes. Two genes with a syntenic relationship were joined by the lines. Additional file 2:
**Phylogenetic tree for** ***Hsf*** **genes of six Roseceae species.** 137 Hsf protein sequences were used, including 29 PbHsfs, 25 MdHsfs, 33 PcHsfs, 17 PpHsfs, 16 FvHsfs, 17 PmHsfs. A, B and C stands for the three major groups of *Hsf* genes. *Hsf* genes were further classified into 15 subgroups (A1, A2, A3, A4, A5, A6, A7, A8, A9, B1, B2, B3, B4, B5, C). The abbreviations of species names are as follows: Pb, Chinese white pear; Md, apple; Pp, peach; Fv, strawberry; Pm, Chinese plum; Pc, European pear. Additional file 3:
**Gene features of** ***Hsf*** **genes in five Rosaceae species.**
Additional file 4:
**Exon-intron structure of** ***Hsfs*** **genes in Chinese white pear, peach, Chinese plum.** Exons are indicated by the yellow boxes. Introns are represented by black lines, and blue boxes represent Untranslated Regions (UTR). Intron phase was showed by 0, 1, 2. The capital letter (A, B, and C) and number after each gene name indicate the subfamily to which it belongs. Additional file 5:
**Exon-intron structures of** ***Hsf*** **genes in strawberry, apple and European pear.** Exons are indicated by the yellow boxes. Introns are represented by black lines, and blue boxes represent Untranslated Regions (UTR). Intron phase was showed by 0, 1, 2. Additional file 6:
**Motif sequences identified by MEME tools in pear Hsfs.**
Additional file 7:
**Orthologous pairs of** ***Hsf*** **genes between any two Rosaceae species.**
Additional file 8:
**Synteny analysis of** ***Hsf*** **genes regions between Chinese white pear and other Roseceae species.**
Additional file 9:
**Ka/Ks ratios of paralogous genes among** ***Hsf*** **gene family in Roseceae.**
Additional file 10:
**The ESTs for putative** ***PbHsf*** **genes.**
Additional file 11:
**The RPKM (reads per kilobase per million) value of** ***Hsf*** **genes expression in Chinese white pear.**

## Abbreviations

WGD: Whole-genome duplication
MYA: Million years ago
DBD: N-terminal DNA binding domain
HR-A/B: Bipartite oligomerization domain
NLS: Nuclear localization signal
NES: Nuclear export signal
CTAD: C-terminal activator domain
HMM: Hidden Markov Model
NJ: Neighbor-joining
ML: Maximum likelihood
RING: Really Interesting New Gene
WD40: Tryptophan-aspartic acid 40
MEME: Multiple EM for Motif Elicitation
PGDD: Plant genome duplication database
ESTs: Expressed sequence tags
GDR: Genome database for Rosaceae
AP2/ERF: APETALA 2/ethylene-responsive element binding factor
MADS: MINICHROMOSOME MAINTENANCE1, AGAMOUS, DEFICIENS and SERUM RESPONSE FACTOR
NBS-LRR: Nucleotide-binding site-leucine-rich repeat
