# Supplementary figures and images for: Genome-wide identification and comparative analysis of the heat shock transcription factor family in Chinese white pear (Pyrus bretschneideri) and five other Rosaceae species
Source: BMC Plant Biol. 2015 Jan 21;15:12. doi: 10.1186/s12870-014-0401-5 (PMC4310194; doi:10.1186/s12870-014-0401-5)

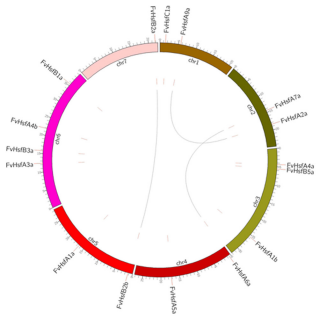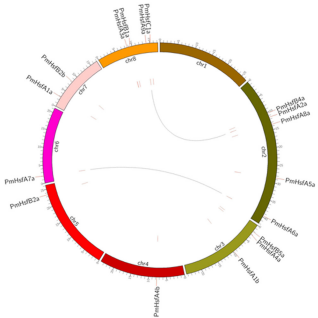

Supplement: Additional file 1: — Location of Hsf genes in strawberry and Chinese plum. Hsf genes in strawberry(FvHsf), and Chinese plum (PmHsf) were mapped on the different chromosomes. Chromosome number is indicated on the inner side and highlighted red short lines in the inner circle correspond to different Hsf genes. Two genes with a syntenic relationship were joined by the lines. [file 12870_2014_401_MOESM1_ESM.pdf]

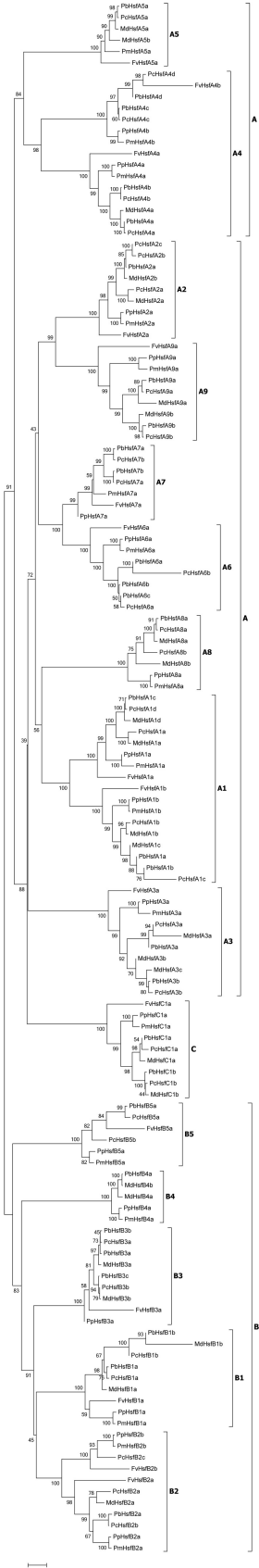

Supplement: Additional file 2: — Phylogenetic tree for Hsf genes of six Roseceae species. 137 Hsf protein sequences were used, including 29 PbHsfs, 25 MdHsfs, 33 PcHsfs, 17 PpHsfs, 16 FvHsfs, 17 PmHsfs. A, B and C stands for the three major groups of Hsf genes. Hsf genes were further classified into 15 subgroups (A1, A2, A3, A4, A5, A6, A7, A8, A9, B1, B2, B3, B4, B5, C). The abbreviations of species names are as follows: Pb, Chinese white pear; Md, apple; Pp, peach; Fv, strawberry; Pm, Chinese plum; Pc, European pear. [file 12870_2014_401_MOESM2_ESM.pdf]

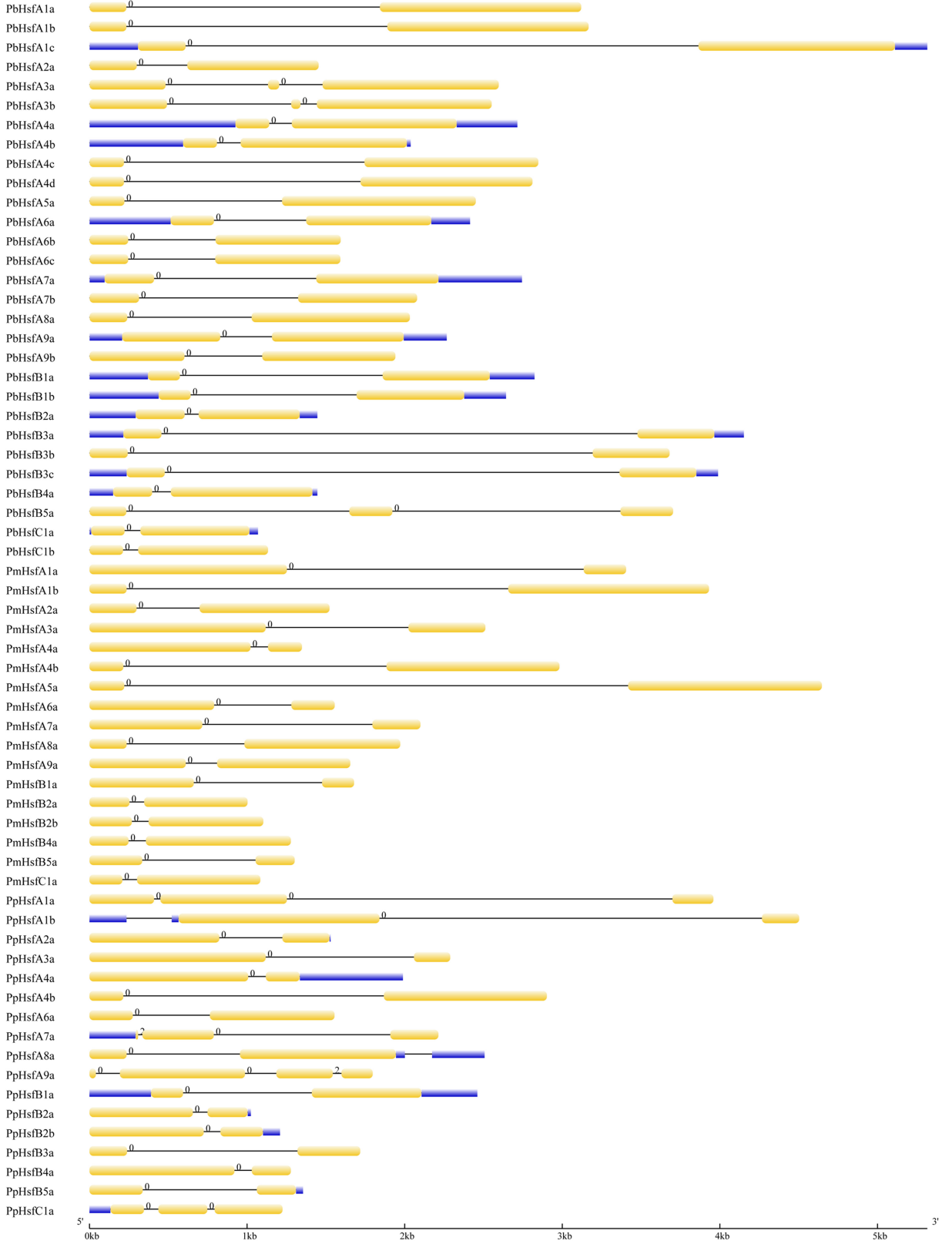

Supplement: Additional file 4: — Exon-intron structure of Hsfs genes in Chinese white pear, peach, Chinese plum. Exons are indicated by the yellow boxes. Introns are represented by black lines, and blue boxes represent Untranslated Regions (UTR). Intron phase was showed by 0, 1, 2. The capital letter (A, B, and C) and number after each gene name indicate the subfamily to which it belongs. [file 12870_2014_401_MOESM4_ESM.pdf]

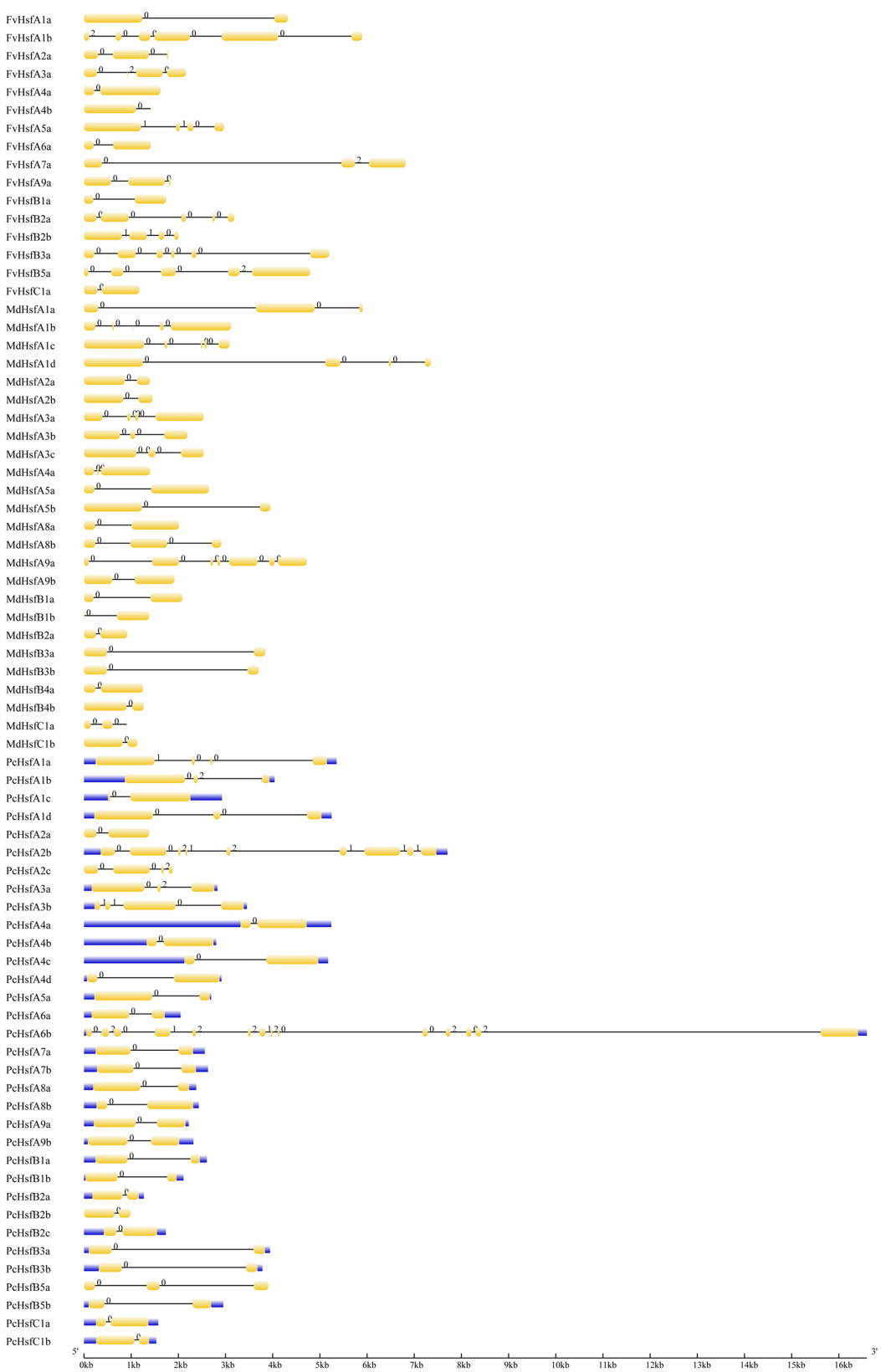

Supplement: Additional file 5: — Exon-intron structures of Hsf genes in strawberry, apple and European pear. Exons are indicated by the yellow boxes. Introns are represented by black lines, and blue boxes represent Untranslated Regions (UTR). Intron phase was showed by 0, 1, 2. [file 12870_2014_401_MOESM5_ESM.pdf]
